# Dystrophinopathy with a *DMD* exon 49–50 deletion in a female patient who developed schizophrenia: An autopsy case

**DOI:** 10.1002/pcn5.70327

**Published:** 2026-04-05

**Authors:** Shusei Arafuka, Youta Torii, Ayako Miwa, Itaru Kushima, Hirotaka Sekiguchi, Hiroshige Fujishiro, Chikako Habuchi, Hiroaki Miyahara, Yosuke Kawai, Ran Wei, Katsushi Tokunaga, Norio Ozaki, Mari Yoshida, Shuji Iritani, Yasushi Iwasaki, Masashi Ikeda

**Affiliations:** ^1^ Department of Psychiatry Nagoya University Graduate School of Medicine Nagoya Aichi Japan; ^2^ Department of Neuropathology, Institute for Medical Science of Aging Aichi Medical University Nagakute Aichi Japan; ^3^ Moriyama General Mental Hospital Nagoya Aichi Japan; ^4^ Department of Psychiatry Okehazama Hospital Fujita Mental Care Center Toyoake Aichi Japan; ^5^ Aichi Psychiatric Medical Center Nagoya Aichi Japan; ^6^ Genome Medical Science Project, Research Institute National Center for Global Health and Medicine Tokyo Japan; ^7^ Pathophysiology of Mental Disorders Nagoya University Graduate School of Medicine Nagoya Aichi Japan; ^8^ Institute for Glyco‐core Research (iGCORE) Nagoya University Nagoya Aichi Japan

**Keywords:** dystrophin, Duchenne muscular dystrophy, intellectual disability, manifesting carrier, schizophrenia

## Abstract

**Background:**

Mutations in *DMD* affect not only muscles but also the brain. Cases of schizophrenia with *DMD* mutations have been described previously. Although female dystrophinopathy often has a milder phenotype, some affected females also have intellectual disabilities and psychiatric disorders. We herein present an integrated account of the clinical course, genetic information, and neuropathological findings of a female patient with dystrophinopathy who developed schizophrenia.

**Case Presentation:**

The patient exhibited a developmental delay, intellectual disability, and schizophrenia, and died at 66 years of age from renal and heart failure. A copy number variation analysis and whole‐genome sequencing revealed a heterozygous deletion in chrX:31774440–31859356 (hg38), encompassing exons 49 and 50 of *DMD* (NM_004006.3). A neuropathological examination showed that the cortical layers were largely preserved; however, in parts of the visual cortex, some neurons were densely arranged in a line across layers IV–V, and single‐neuronal heterotopias were observed in the subependymal regions around the lateral ventricles and within the white matter of the middle frontal gyrus. These findings suggest focal abnormal brain development that was milder than that reported in Duchenne muscular dystrophy, which may in part reflect the reduced and mosaic expression of dystrophin due to X‐chromosome inactivation.

**Conclusion:**

The neuropathological findings in this case and in previous studies of Duchenne muscular dystrophy partially overlap with those of schizophrenia. To elucidate the pathogenesis and relationship between schizophrenia and *DMD* mutations, clinical and research attention should be directed toward the coexistence of both disorders.

## BACKGROUND

Duchenne muscular dystrophy (DMD) and Becker muscular dystrophy (BMD) are severe muscle diseases caused by mutations in *DMD* located in the Xp21 region.^1^, ^2^ *DMD* encodes various dystrophin protein (Dp) isoforms in both muscle and brain tissues.^1^, ^3^, ^4^, ^5^, ^6^ Brain impairment caused by *DMD* mutations is thought to induce intellectual disability and various psychiatric symptoms.^5^, ^6^ Cases of schizophrenia with DMD and BMD have been described previously.^7^, ^8^, ^9^ Although *DMD* mutations follow an X‐linked recessive inheritance pattern, some females with dystrophinopathy develop various symptoms.^10^, ^11^ Up to 19% of females with dystrophinopathy exhibit muscle weakness,^12^, ^13^, ^14^, ^15^ and some cases of dilated cardiomyopathy have been reported.^14^, ^15^ In addition to muscular symptoms, cognitive and psychiatric manifestations have also been described. For example, one study reported that learning disabilities were present in 5 of 26 females with dystrophinopathy, and intellectual disability was present in 2 of 26.^10^ In another report, 7 of 9 females with dystrophinopathy had intellectual disability, and 5 of 9 had at least one psychiatric disorder.^11^ The reported psychiatric disorders included oppositional defiant disorder, adjustment disorder, eating disorder, attention deficit hyperactivity disorder, bipolar disorder, depression, and autistic features. *DMD* mutations can affect the central nervous system even in female dystrophinopathy. Despite the importance of investigating female dystrophinopathy to understand the pathogenesis, to the best of our knowledge, all prior autopsy cases were male, and there have been no neuropathological reports of female dystrophinopathy. This study reports the first autopsied case of a female with dystrophinopathy who developed schizophrenia and provides valuable insights into the phenotype through a detailed clinical course, genetic analysis, and neuropathological findings.

## CASE PRESENTATION

### Clinical assessments

Multiple expert psychiatrists who were blinded to the neuropathological diagnosis retrospectively evaluated the clinical diagnosis based on the patient's medical records.

### Genetic analyses

Genomic DNA was extracted from a blood sample. We used NimbleGen 720k Whole‐Genome Tiling arrays (Roche NimbleGen) for copy number variation (CNV) analysis. CNV calls were generated using Nexus Copy Number (ver. 9.0, BioDiscovery). The high accuracy of CNV calls from this array, exceeding 96%, has been previously confirmed.^16^ The deletion was confirmed by whole‐genome sequencing using a NovaSeq. 6000 system (Illumina). Library preparation was performed using the TruSeq DNA PCR‐Free Kit (Illumina). Sequencing was performed with paired‐end reads targeting a 30× depth. Raw sequence data (FASTQ files) were obtained from the contract laboratory, followed by alignment with the human reference genome (GRCh38) using the BWA‐mem algorithm implemented in Parabricks 3.6 (Nvidia). CNV calling was performed using CNVkit.^17^

### Neuropathological techniques

The brain was fixed in a 20% buffered formalin solution and embedded in paraffin. Macroscopic observations and tissue preparation were performed according to previously described standard practices.^18^ Sections of 9 μm thickness were stained with hematoxylin and eosin, Klüver–Barrera, or Gallyas–Braak, and sections of 4.5 μm thickness were used for immunohistochemistry (Table 1).

**Table 1:** The primary antibodies used for immunohistochemistry.

Name of Antibody	Supplier	Clonality	Source	Dilution	Unmasking
Anti‐phosphorylated tau	Thermo Fisher Scientific	Monoclonal	Mouse	1:4000	Heat antigen retrieval and formic acid
Anti‐beta‐amyloid (11‐28)	Immuno‐Biological Laboratories	Monoclonal	Mouse	1:1000	Formic acid
Anti‐α‐synuclein	Sigma Aldrich	Polyclonal	Rabbit	1:20000	Heat antigen retrieval and formic acid
Anti‐phosphorylated α‐synuclein	Wako Pure Chemical Industries	Monoclonal	Mouse	1:6000	Heat antigen retrieval and formic acid
Anti‐phosphorylated transactive response DNA‐binding protein 43 kDa	Cosmobio	Polyclonal	Rabbit	1:4000	Heat antigen retrieval and formic acid
Anti‐phosphorylated neurofilament	Covance	Monoclonal	Mouse	1:5000	Heat antigen retrieval
Anti‐glial fibrillary acidic protein	Dako	Monoclonal	Mouse	1:500	Heat antigen retrieval

### Clinical summary

The patient presented with developmental delays, particularly in language skills, and attended a special education class for intellectual disabilities. Detailed information about her father was limited to a history of alcohol dependence. Her mother had no confirmed history of psychiatric or physical illnesses. Her older brother developed muscle weakness and psychiatric symptoms during childhood and died at age 25. A congenital muscle disease was suspected in the older brother, according to the medical record. Her younger brother lived in a nursing home at the time of the patient's death, primarily because of renal failure requiring hemodialysis and difficulty living alone; no muscular symptoms or diagnosis were documented. Genetic testing was not performed on the siblings or parents. At 22 years of age, she began to experience persecutory delusions and insulting verbal auditory hallucinations, which led to a suicide attempt and hospitalization. Although the patient declined detailed neuropsychological testing, no visual hallucinations or visual impairment suggestive of occipital dysfunction were documented. She exhibited violent and disorganized behavior with poor emotional control, such as stripping naked on the ward or lying on the floor. Following the onset of psychiatric symptoms, her social functioning declined, leaving her unable to work in a food factory or manage her finances independently. Multiple psychiatrists diagnosed the patient with schizophrenia. She further presented with multiple physical complications, including muscle fatigue, urinary incontinence, and persistently elevated serum creatine kinase levels (range: 300–800 U/L). Although adequate doses of antipsychotic medication provided partial relief, she frequently discontinued treatment, leading to repeated relapses. Following her mother's death, she could no longer live alone and remained hospitalized until her death at age 66 due to renal and heart failure. Although she began to experience occasional falls from around 60 years of age, she was able to walk independently and participate in exercise programs until two months before her death. The DMD mutation could have contributed to the heart failure, but a detailed cardiac evaluation was not performed.

### Genetic findings

CNV analysis revealed a heterozygous deletion on chromosome X at 31774440–31859356 (hg38), encompassing exons 49 and 50 of *DMD* (transcript NM_004006.3). The deletion was confirmed by whole‐genome sequencing using GRCh38 as a reference.

### Neuropathological findings

**Figure 1:**
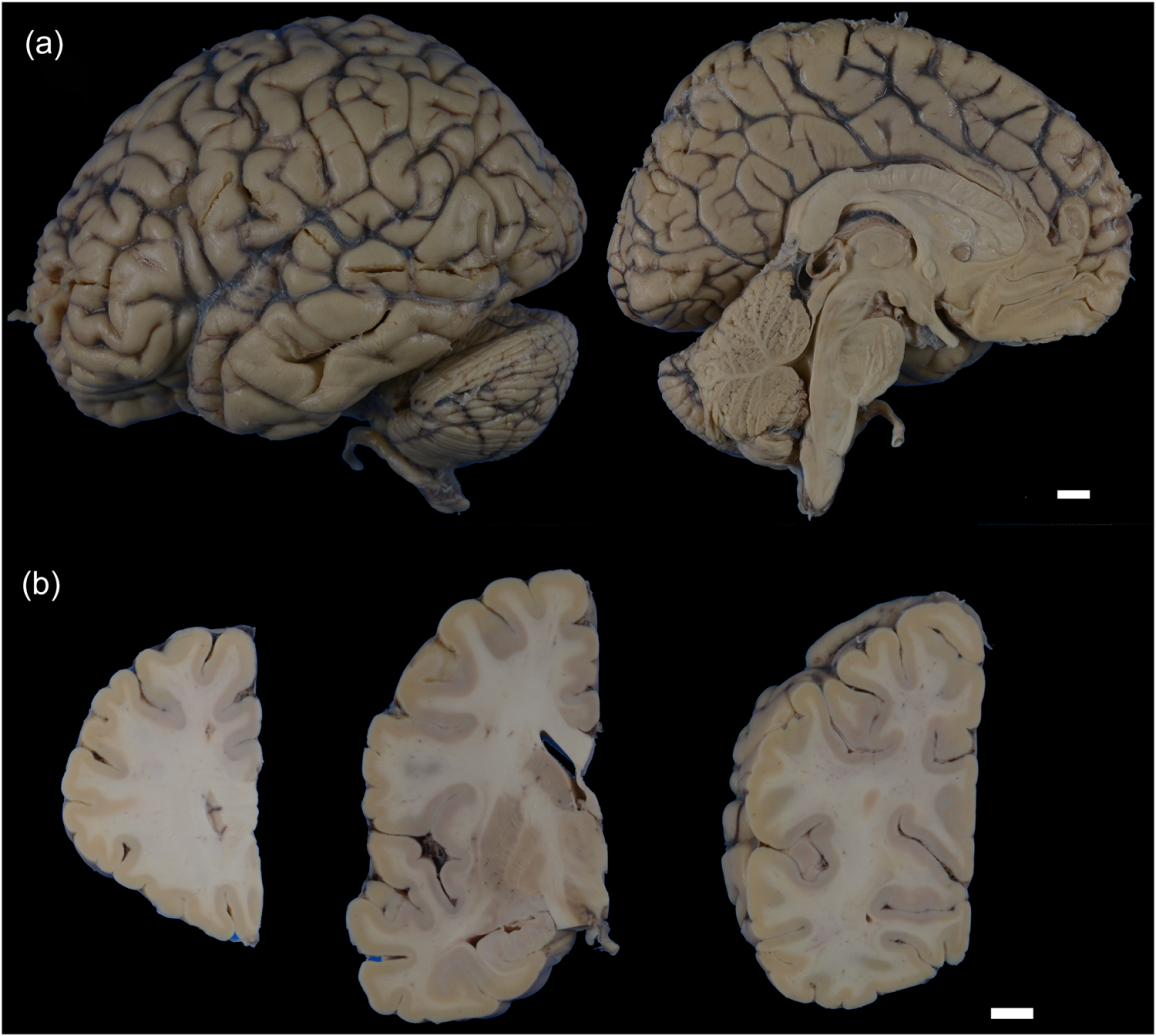
Macroscopic findings. The left cerebral hemisphere, after formalin fixation, shows no focal atrophy (a). Coronal sections of the cerebrum after formalin fixation reveal no obvious abnormalities (b). The left coronal sections pass through (b, Left) the edge of the anterior horn of the lateral ventricle; (b, Middle) the anterior commissure, striatum, and hippocampus; and (b, Right) the calcarine sulcus. Scale bar: 10 mm (a, b).

An autopsy was performed approximately 1.5 h postmortem. The brain weighed 1034 g. Macroscopic observations revealed no focal cerebral atrophy or visible abnormalities in the cerebellum, brainstem, or spinal cord (Figure 1a,b).

**Figure 2:**
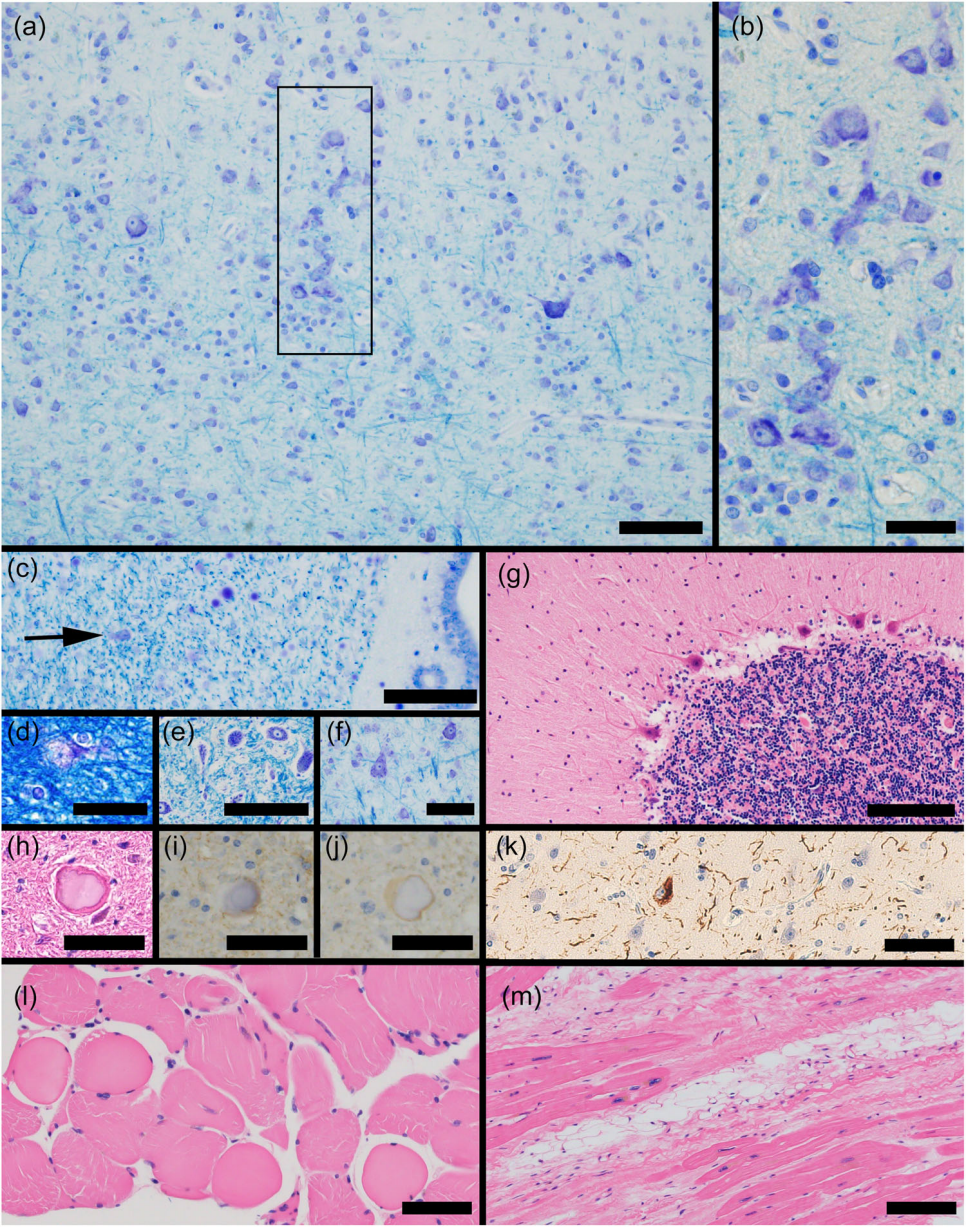
Microscopic findings. Klüver–Barrera staining shows some neurons densely arranged in a line across layers IV–V in parts of the visual cortex (a, b). Klüver–Barrera staining shows single‐neuronal heterotopia observed in the subependymal regions around the lateral ventricles (c, as indicated by the arrow) and in the white matter of the middle frontal gyrus (d). Anterior horn neurons of the spinal cord (e) and Betz cells in the precentral gyrus (f) are preserved. The three layers of the cerebellum and Purkinje cells are well‐preserved (g). Several eosinophilic structures are found in the gracile nucleus (h) with negative immunostaining of anti‐glial fibrillary acidic protein (i) and anti‐phosphorylated neurofilament (j). Neurofibrillary tangles and neuropil threads are observed in a limited region of the transentorhinal cortex (k). Skeletal muscle fibers display variability in size, with some showing a rounded morphology (l). Interstitial spreading and fibrosis of the myocardial fibers are observed (m). Scale bars: 100 μm (e, g, m), 50 μm (a, c, f, h–l), and 20 μm (b, d).

A microscopic examination revealed that the layered structure of the cerebral cortex was preserved in most of the cortical regions. However, in parts of the visual cortex, some neurons were densely arranged in a line across layers IV–V (Figure 2a,b). This abnormal arrangement did not follow the layered structure of the cortex. However, no comparable abnormalities of the layered structure were found in other examined cortices. Single‐neuronal heterotopias (misplaced neurons) were sporadically observed in the deep white matter of the cerebrum, including the subependymal regions around the lateral ventricles, the deepest part of the cerebrum (Figure 2c). Single‐neuronal heterotopias were relatively prominent in the middle frontal gyrus (Figure 2d). The hippocampal CA1–CA4 and subiculum were well preserved. No abnormalities were observed in the amygdala, cingulate gyrus, nucleus accumbens, nucleus basalis of Meynert, putamen, caudate nucleus, globus pallidus, thalamus, or subthalamic nucleus. The anterior horn motor neurons (Figure 2e) and Betz cells (Figure 2f) were preserved, with no significant abnormalities in the pyramidal tract. The three layers of the cerebellum were preserved, and Purkinje cell loss was minimal (Figure 2g). Mild arteriosclerosis and atherosclerosis were observed in the cerebral vasculature. A few microinfarcts were also identified in the cortex. Klüver–Barrera staining revealed well‐stained cerebral white matter with no evidence of myelin pallor. Overall, the vascular pathology was minimal. Several eosinophilic structures were observed in the gracile nucleus of the brainstem (Figure 2h). These structures were not stained by anti‐glial fibrillary acidic protein or anti‐phosphorylated neurofilament (Figure 2i,j), suggesting that they did not represent astrogliosis or axonal swelling (spheroids), and the significance of these structures was unclear. Age‐related pathological changes were less pronounced, as follows: neurofibrillary tangle Braak stage,^19^ 1 (Figure 2k); Thal phase,^20^ 0; Consortium to Establish a Registry for Alzheimer's Disease Neuritic Plaque Score,^21^ 0; amyloid angiopathy (–), phosphorylated‐α‐synuclein‐positive structures (–), argyrophilic grain (–), tufted astrocyte (–), astrocytic plaque (–), and phosphorylated transactive response DNA‐binding protein 43 kDa‐positive structures (–). Skeletal muscle fibers displayed variability in size, with some showing rounded morphology (Figure 2l). However, the overall degree of muscle abnormality was mild, consistent with the patient's preserved motor function. Interstitial spreading and fibrosis of the myocardial fibers were also observed as a result of heart failure (Figure 2m).

## DISCUSSION AND CONCLUSION

### Pathological findings suggestive of brain maldevelopment in DMD mutations

Certain dystrophin isoforms are expressed in the central nervous system, and their dysfunction may affect brain development.^5^, ^6^ This female with dystrophinopathy exhibited typical symptoms of dystrophinopathy, including muscle symptoms, urinary incontinence, persistently elevated serum creatine kinase levels, heart failure, and delayed intellectual development,^1^, ^2^, ^22^ suggesting that this *DMD* deletion had a significant impact on her condition. The patient's developmental and intellectual disabilities support the underlying abnormal brain development. The risk of intellectual disability has been linked to the cumulative involvement of brain‐expressed dystrophin isoforms, with reported associations particularly for fetal brain‐predominant Dp140.^5^, ^23^, ^24^, ^25^ The exon 49–50 deletion is considered out‐of‐frame and may potentially affect Dp427, Dp260, and Dp140 in this case.^26^, ^27^ While previous neuropathological reports highlight the lack of morphological abnormalities in most brains with *DMD* mutations,^6^, ^28^ some patients with *DMD* mutations show neuropathological findings suggestive of brain maldevelopment. Several reports have noted neuronal heterotopias in DMD patients.^29^, ^30^, ^31^, ^32^ Abnormal multifocal hamartomatous glial nodules, presumed to have developed during early brain development, were reported in the cortex of a patient with DMD and intellectual disability.^32^ In autopsy reports of multiple DMD patients, heterotopias were more remarkable and deeper in DMD patients with comorbid intellectual disability than in those without.^29^ In the case of DMD with developmental delay and intellectual disability, in addition to heterotopia, the brain shows abnormal development with disrupted cortical layering.^30^ A quantitative analysis of Golgi staining of the visual cortex in three patients revealed a reduction in the length and branching of dendrites of neurons, suggesting abnormal development.^31^ Despite methodological differences, the present case and the cases reported by Jagadha and Becker indicate the presence of visual cortex maldevelopment.^31^ The presence of sporadic single‐neuronal heterotopias in the deep brain regions of our case was consistent with previous reports, suggesting abnormal neuronal migration. These abnormal findings in our case were considered less pronounced than those reported in earlier cases of DMD. This may be attributable in part to our patient being a female with dystrophinopathy. *DMD* mutations follow an X‐linked recessive inheritance pattern, resulting in milder phenotypes in female dystrophinopathy relative to those observed in DMD. Dystrophin expression is expected to be mosaic rather than completely absent due to *X*‐chromosome inactivation,^33^ which may cause functional deficiency in specific tissues or cell populations, potentially leading to focal neurodevelopmental abnormalities. This focal and nonuniform distribution of abnormalities is consistent with this perspective.

### Possible common pathological findings between DMD mutations and schizophrenia

Consistent with the neurodevelopmental disorder hypothesis,^34^, ^35^ schizophrenia is also thought to develop from abnormal brain development. The pathological findings presented in previous reports in DMD patients and our case share similarities with those described in the postmortem studies of schizophrenia. Abnormalities in the layered structure of the entorhinal cortex^36^, ^37^, ^38^; increased density and distribution of white matter heterotopic neurons^39^, ^40^; and synaptic, axonal, and dendritic abnormalities^41^, ^42^, ^43^ have been reported in neuropathological studies of schizophrenia. However, brain phenotypes are heterogeneous, and overt morphological abnormalities are not consistently observed.^41^ Dystrophinopathy can be considered a primary neurodevelopmental disorder driven by a single‐gene loss‐of‐function, whereas schizophrenia is a multifactorial condition. Accordingly, morphological findings may be interpreted as indications of vulnerability to psychiatric symptoms rather than evidence of a shared mechanism. Nevertheless, a molecular overlap may exist; for example, Dp427 loss may alter GABA_A_ receptor‐mediated inhibition,^44^, ^45^ suggesting a possible overlap with GABAergic dysfunction in schizophrenia.^46^ Further studies are warranted to clarify the relationship between dystrophinopathy and schizophrenia at both the morphological and molecular levels.

### The importance and difficulties of focusing on schizophrenia and DMD mutations

Elucidating the pathogenesis of schizophrenia from the perspective of *DMD* mutations can be a promising approach. However, data on the relationship between schizophrenia and *DMD* mutations remain limited. DMD patients have a median life expectancy of approximately 28.1 years,^47^ whereas the mean age of schizophrenia onset is 25.08 ± 7.27 years in males and 27.81 ± 8.92 years in females.^48^ Some patients with DMD may not live long enough to be diagnosed with schizophrenia. Additionally, patients with DMD often have comorbid intellectual disabilities,^49^, ^50^ which could hinder these patients from externalizing the typical psychiatric symptoms of schizophrenia, such as hallucinations and delusions. Females with dystrophinopathy may also remain undetected because of mild symptoms. To elucidate the pathogenesis of schizophrenia and *DMD* mutations, clinical and research attention should be directed toward the coexistence of both disorders.

## AUTHORS CONTRIBUTIONS

*Study design*: Shusei Arafuka, Youta Torii, Itaru Kushima, Norio Ozaki, Shuji Iritani and Masashi Ikeda. *Clinical data acquisition and evaluation*: Ayako Miwa, Hirotaka Sekiguchi, Hiroshige Fujishiro, Chikako Habuchi and Shuji Iritani. *Genetic analysis*: Itaru Kushima, Yosuke Kawai, Ran Wei, Katsushi Tokunaga, and Masashi Ikeda. *Neuropathological evaluation*: Shusei Arafuka, Hiroaki Miyahara, Mari Yoshida, and Yasushi Iwasaki. *Drafting the original manuscript*: Shusei Arafuka, Youta Torii, and Itaru Kushima. *Critical revision*: Hiroshige Fujishiro, Norio Ozaki, Shuji Iritani, Mari Yoshida, Yasushi Iwasaki, and Masashi Ikeda. All authors reviewed and approved the final manuscript.

## CONFLICT OF INTEREST STATEMENT

Norio Ozaki received research support or speaker honoraria from or has served as a joint researcher with, or a consultant to, Sumitomo Pharma, Eisai, Otsuka, KAITEKI, Mitsubishi Tanabe, Shionogi, Eli Lilly, Mochida, DAIICHI SANKYO, TSUMURA, Takeda, Meiji Seika Pharma, Kyowa, EA Pharma, Viatris, Kyowa Kirin, MSD, Janssen, Yoshitomi, Ricoh, Taisho, and Nippon Boehringer Ingelheim, outside the submitted work. Masashi Ikeda has received research support or speaker honoraria from Sumitomo Pharma, Eisai, Otsuka, Tanabe Mitsubishi, Mochida, Takeda, Meiji Seika Pharma, EA Pharma, Viatris, MSD, Janssen, Lundbeck, and Yoshitomi outside the submitted work. The remaining authors declare no conflicts of interest.

## ETHICS APPROVAL STATEMENT

This study was approved by the Ethics Review Committee of the Nagoya University Graduate School of Medicine. The autopsy was performed in accordance with the “Corpse Autopsy and Preservation Law.”

## PATIENT CONSENT STATEMENT

After the patient's death, written consent for publication was obtained from the proxy.

## CLINICAL TRIAL REGISTRATION

N/A.

## Data Availability

Data supporting this study are available from the corresponding author upon reasonable request.
